# A novel technique for the impression, model fabrication and provisionalization of pinlays

**DOI:** 10.15171/joddd.2018.012

**Published:** 2018-03-14

**Authors:** Les Kalman, Quincy Izabela Sofowora

**Affiliations:** ^1^Assistant Professor, Restorative Dentistry, Faculty Lead, Dental Outreach, Schulich School of Medicine & Dentistry, Western University, Lon-don, Ontario, Canada; ^2^Dental graduate, Wroclaw Medical University, Wrocław, Poland

**Keywords:** CAD/CAM, dental pins, impression alternative, onlay

## Abstract

Indirect non-metal restorations restore the function and esthetics of severely carious or broken teeth. This report explores a novel approach for the impression, model fabrication and provisionalization of a pin reinforced-onlay (pinlay) preparation. A dentoform molar was prepared for a pinlay indirect restoration. An impression, model and a provisional were fabricated with the employed pins and determined to be clinically acceptable. The pinlay may offer the patient and clinician another treatment option for oral rehabilitation.

## Introduction

Direct restorations aim to immediately rebuild a tooth. There are clinical situations where the indirect restoration is the ideal treatment option for the patient. The typical workflow^4^ for indirect restoration is tooth preparation, followed by an impression and provisionalization. The impression is sent to a laboratory for the fabrication of the model and subsequent restoration and the patient is seen for a second appointment for cementation. With technological advances, there has been the opportunity for one-appointment in-office indirect restoration fabrication through CAD/CAM.^5^ However, this approach requires significant experience, clinical skill and a schedule that permits the workflow. Single visit indirect restorations are very difficult in an academic setting, as students lack the experience and efficiency for the one-appointment workflow.

**Figure 1:**
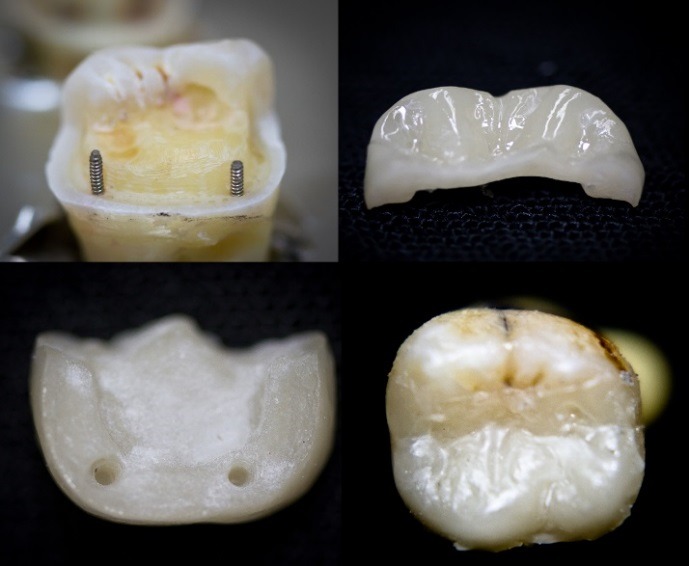
In vitro e.max press pinlay.

Although esthetic materials (zirconia and e.max) possess very high physical strength,^6^ the retention of the restoration on the tooth is dependent upon the preparation and bond strength.^7^ In addition, operator error, contamination and parafunction can lead to failure.^8^ Pinlays (Figure 1) have been explored in the dental literature^9^ and a recent study compared the strength of conventional onlays compared to pin-reinforced onlays (pinlays). Although the results showed no statistical difference in fracture resistance between the two groups, the highest fracture resistance was found in a pin-retained e.max onlay.

This report explores a novel approach for the impression, model fabrication and provisionalization of a pin-reinforced onlay (pinlay) preparation. The concept behind the investigation is that the treatment would be a two-step process, where the tooth is prepared and provisionalized and the restoration is delivered during a second appointment.

## Methods

**Figure 2:**
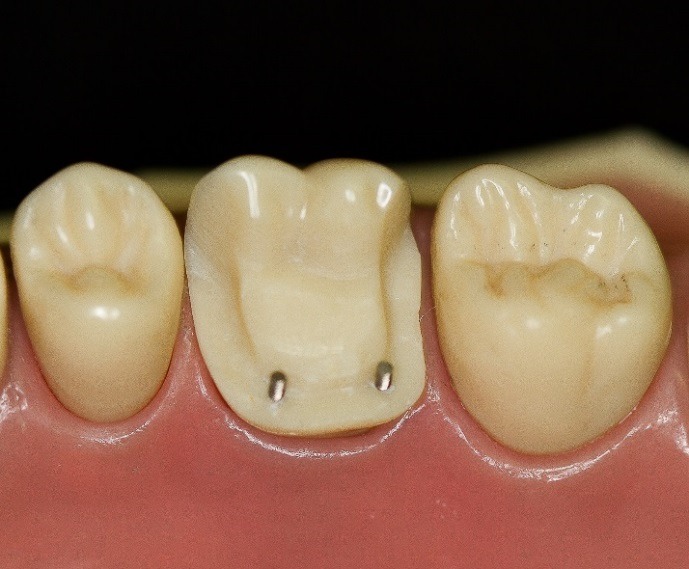
Onlay preparation on tooth #16 with 2 retentive pins

**Figure 3:**
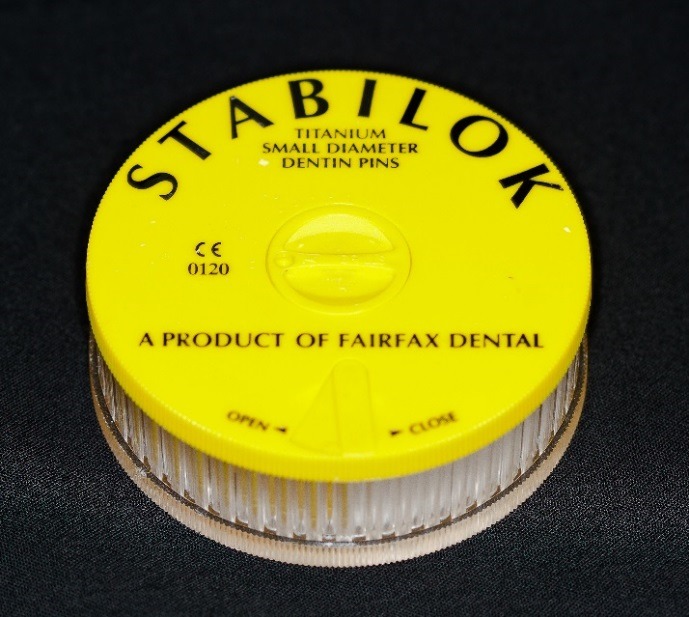
Stabilok retentive pins.

**Figure 4:**
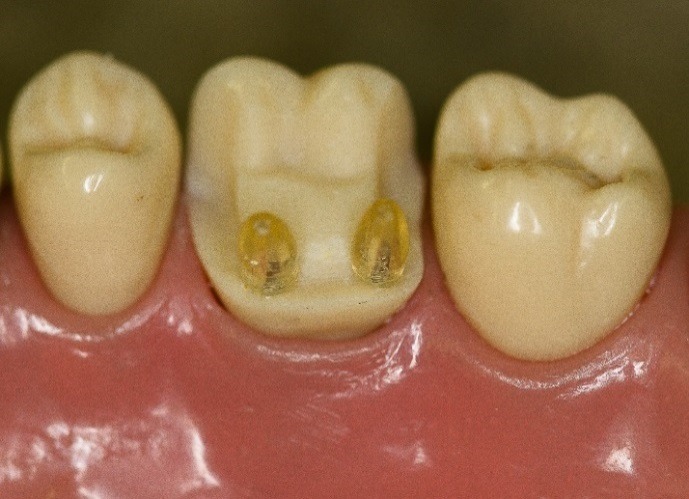
Retentive pin sheaths placed over pins.

An upper molar (tooth #16) dentoform tooth (Columbia Dentoform, Long Island, USA) was used to prepare a four-surface mesial-occlusal-distal-lingual onlay preparation (Figure 2), employing 0.6 mm titanium pins (Figure 3) (Fairfax Dental, London, England) at the mesial‒lingual and distal‒lingual line angles. Pin sheaths (Figure 4) were used to cover the retentive pin.****

**Figure 5:**
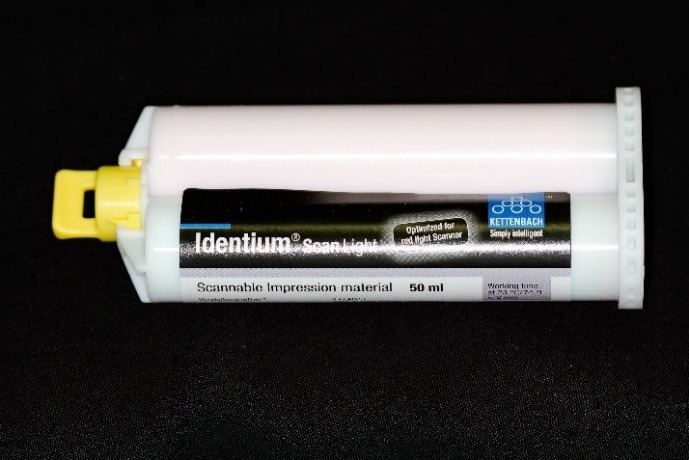
Identium light body impression material.

**Figure 6:**
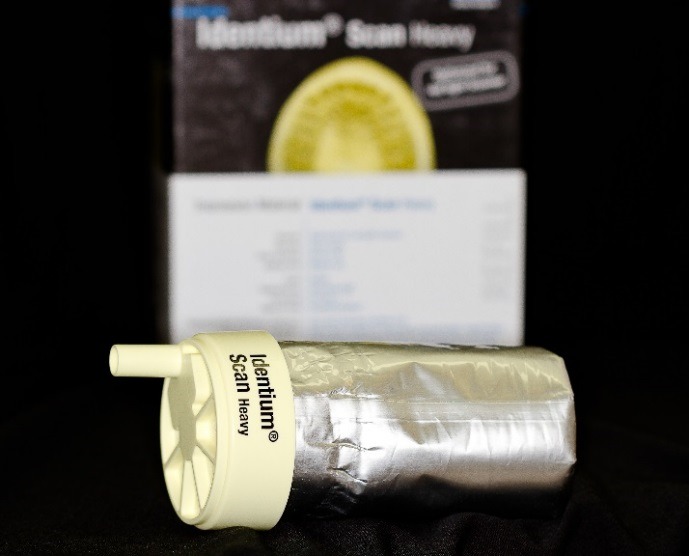
Identium heavy body impression material.

**Figure 7:**
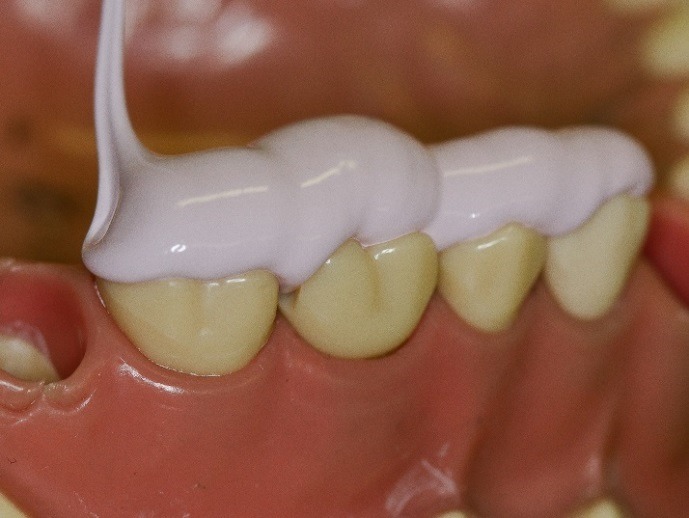
Light body dispensed onto onlay preparation.

**Figure 8:**
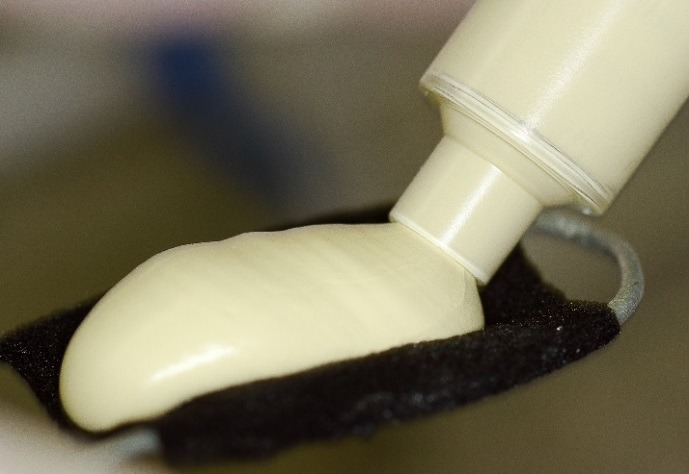
Heavy body dispensed onto triple tray.

**Figure 9:**
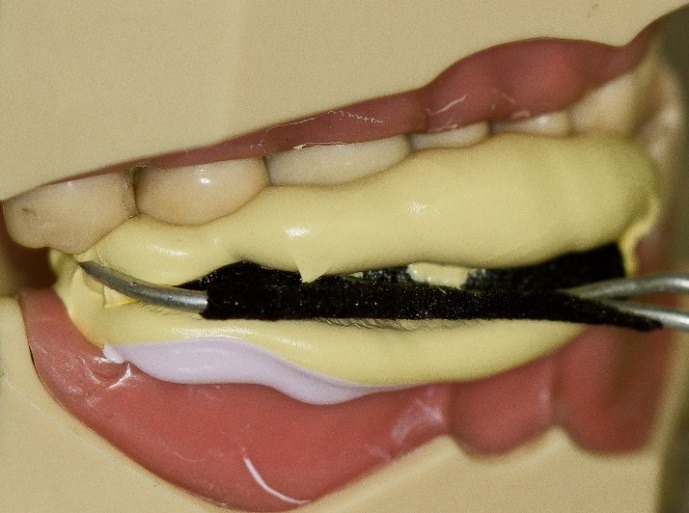
Triple impression technique.

Vinylsiloxanether scan impression material (Kettenbach, Eschenburg, Germany) was used for the impression (Figures 5 and 6). The light body was dispensed onto the occlusal surfaces of the dentoform teeth (Figure 7) and the heavy body was dispensed on the triple tray (Tres Perfect: Research Driven, Kilworth, Canada) (Figure 8).  The triple tray was seated accordingly and the maxillary and mandibular teeth were brought into maximum intercuspation (Figure 9) following manufactures instructions for 2 minutes and 30 seconds.

**Figure 10:**
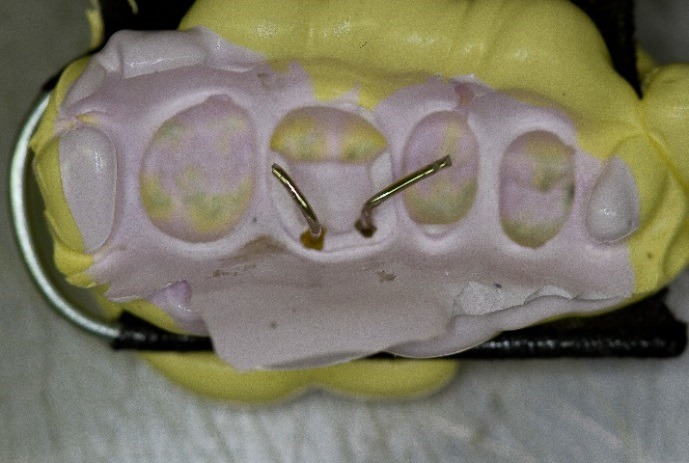
Paper clips placed into pin holes.

**Figure 11:**
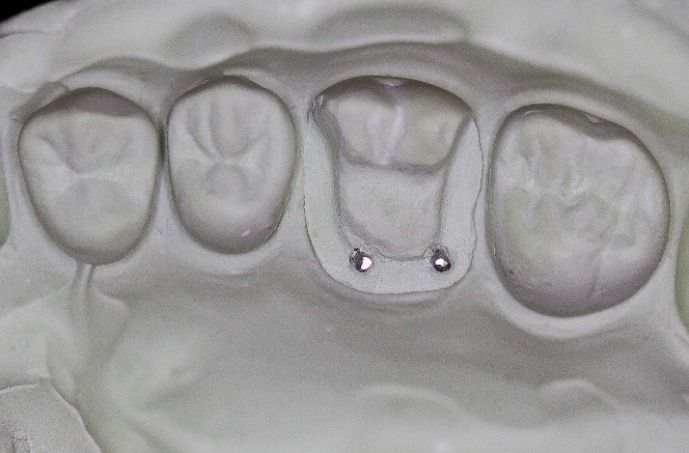
Master cast.

The dentoform teeth were disengaged and the triple tray impression was removed. The sheaths facilitated the removal from the retentive pins. A standard paper clip was fabricated into an L shape and inserted into the sheaths (Figure 10).  The impression was poured with Jade Stone (Whip Mix, Louisville, USA) and the paper clips represented the retentive pin locations (Figure 11). This represented the final cast of the pinlay preparation.

**Figure 12:**
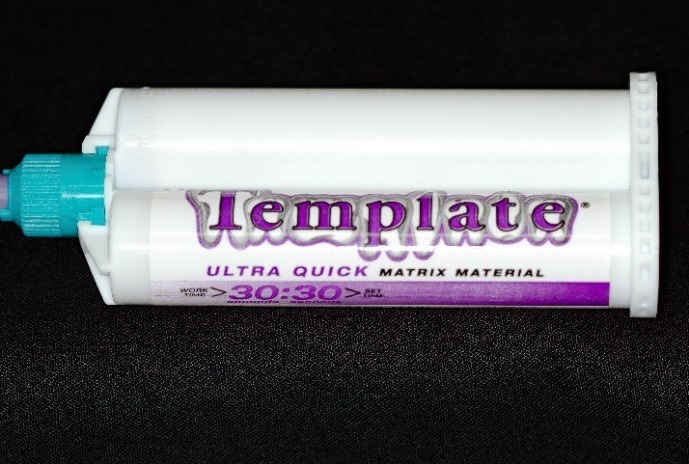
Provisional matrix material.

**Figure 13:**
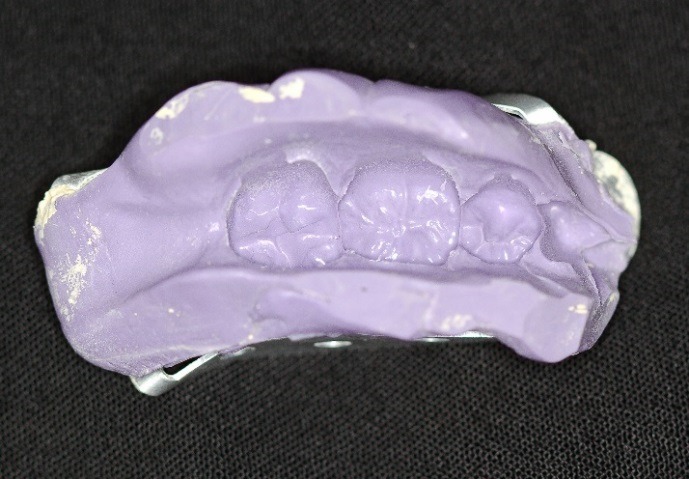
Provisional matrix.

**Figure 14:**
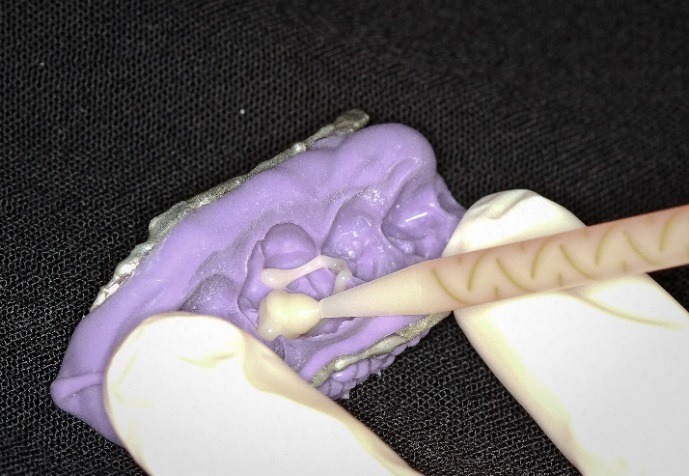
Cling II dispensedinto the provisional matrix.

**Figure 15:**
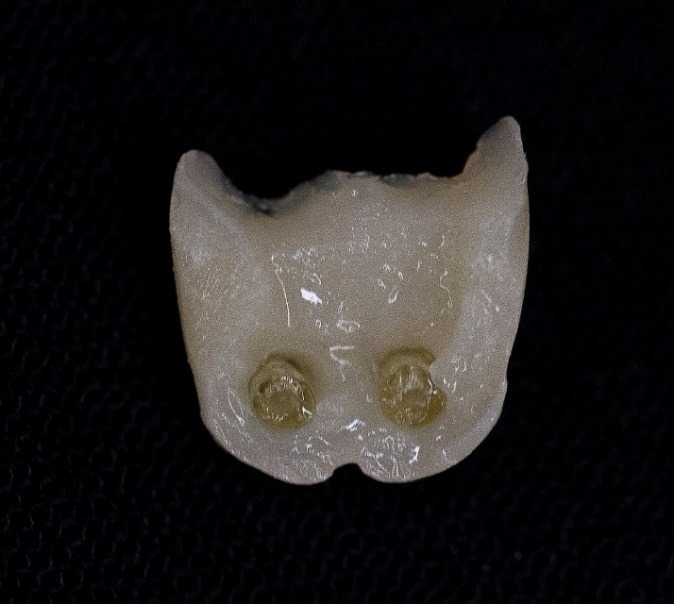
Provisional: internal aspect with sheaths.

**Figure 16:**
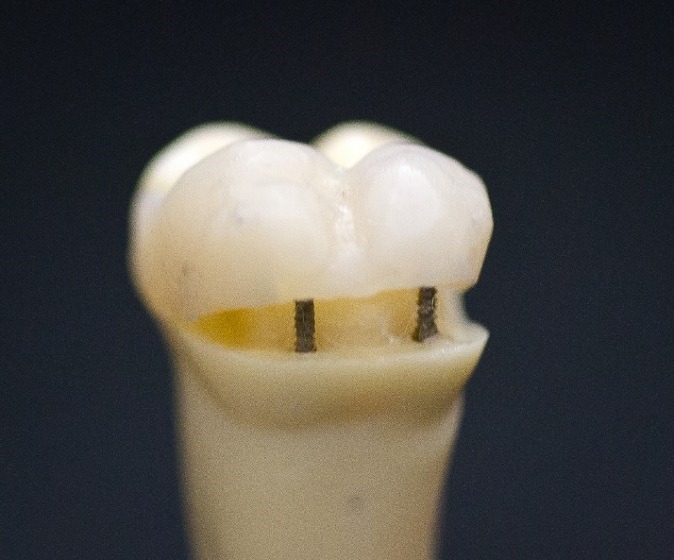
Provisional shaped, shaped and assessed.

**Figure 17:**
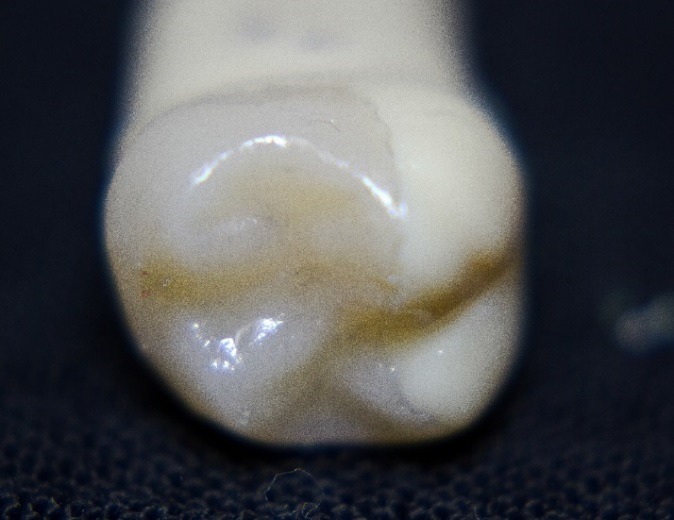
Provisional cemented onto pinlay.

Template (Figure 12) (Clinician’s Choice, New Milford, USA) and Temp tray (Figure 13) (Clinician’s Choice, New Milford, USA) were utilized to create a provisional matrix prior to the pinlay preparation.  The matrix was loaded with bisacryl temporary composite (Figure 14) (Clinician’s Choice, New Milford, USA) and placed over the dentoform pinlay preparation with newly placed pin sheaths. The material was polymerized for three minutes and removed (Figure 15).  The provisional was shaped, polished and assessed for fit and form on the pinlay preparation (Figure 16).  The sheaths remained in the provisional to permit repeated placement and removal. A non-eugenol, polycarboxylate resin temporary cement (Clinician’s Choice, New Milford, USA) was employed to cement the provisional onto the dentoform pinlay preparation (Figure 17).

## Results

The PVS impression was clinically acceptable in capturing all the aspects of the preparation without omissions or artifacts. The paper clips could be successfully placed into the pin hole without difficulty. The Stabilok titanium dentinal pins (manufacturer) were .021 inches or 0.5334 mm in diameter. The paper clips were 0.88 mm in diameter. The larger diameter paper clip ensured that the final proposed restoration would seat around the dentinal pins. It would be interesting to accurately assess the cement film thickness that would encompass the pins.

The cast was clinically acceptable upon visual inspection and captured the dentoform pin preparation adequately. Measurement of the dentform versus cast pin distance was negligible and within the value of the pin sheath thickness. The provisional was assessed to be clinically acceptable, as it sealed the preparation, covered the pins and restored morphology. Evaluation was based on the Schulich criteria utilized for the assessment of impressions and casts for laboratory submission and provisional quality based on clinical criteria.

## Discussion

This preliminary investigation explored a novel approach for the impression and provisionalization of pinlays. Further research is required with a larger sample size and quantitative data to measure variations and distortion from the original to the cast. A clinical assessment would prove valuable to assess variations between the patient and the cast.

As technological advances continue at a tremendous pace, there may be other approaches for the fabrication of indirect pinlays. Additive manufacturing in metal (chromium-cobalt) could be a viable cost-effective option for indirect restorations for those with limited financial resources. Additive manufacturing in porcelain could also present an esthetic option for fabrication.

## Conclusion

This report provides a novel in vitro technique for the impression, model and provisionalization as associated with a two-step pinlay indirect restoration. Indirect restorations remain an important clinical option for the rehabilitation of form and function for severely carious and broken teeth. The pinlay may provide the patient and clinician with another restorative treatment option for the improvement of oral health.

## Additional File

Additional File Video demonstrating a novel technique for the impression, model and provisionalization of pinlays. This material (mp4 file) is available online.

## Acknowledgments

None.

## Competing interests

The authors declare that they have no competing interests with regards to authorship and/or publication of this paper.
